# Methyl 2-(4-chloro-3,5-dinitro­benz­amido)­acetate

**DOI:** 10.1107/S1600536811050446

**Published:** 2011-11-30

**Authors:** Xiang-Xiang Wu, Xue-Fen Wu, Yi-Min Hou, Seik Weng Ng, Edward R. T. Tiekink

**Affiliations:** a Henan University of Traditional Medicine, Zhengzhou 450008, People’s Republic of China; b Department of Chemistry, University of Malaya, 50603 Kuala Lumpur, Malaysia; c Chemistry Department, Faculty of Science, King Abdulaziz University, PO Box 80203 Jeddah, Saudi Arabia

## Abstract

The title mol­ecule, C_10_H_8_ClN_3_O_7_, is twisted with the dihedral angle between the amide and benzene ring being 38.75 (11)°. The C—N—C—C torsion angle between the amide and acetyl groups is −150.1 (2)°. Finally, each nitro group is twisted out of the plane of the benzene ring to which it is connected [O—N—C—C torsion angles = 34.0 (3) and −64.5 (3)°]. Linear supra­molecular chains along [010] and mediated by N—H⋯O hydrogen bonds between successive amide groups dominate the crystal packing. The chains are consolidated into the three-dimensional structure by C—H⋯O contacts.

## Related literature

For biological and crystal engineering studies of related compounds, see: Liu *et al.* (2009 ▶); Eissmann & Weber (2011 ▶).
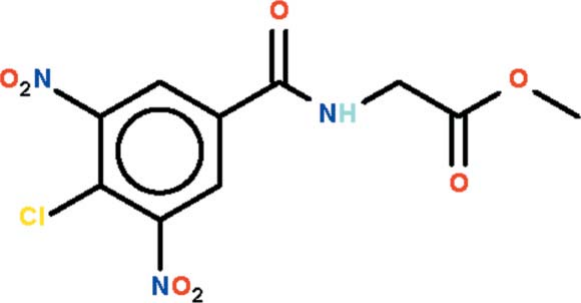


## Experimental

### 

#### Crystal data

C_10_H_8_ClN_3_O_7_
*M* *_r_* = 317.64
Orthorhombic, 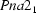

*a* = 14.5219 (5) Å
*b* = 4.7949 (2) Å
*c* = 18.5368 (6) Å
*V* = 1290.74 (8) Å^3^
*Z* = 4
Mo *K*α radiation
μ = 0.34 mm^−1^
*T* = 100 K
0.30 × 0.20 × 0.10 mm

#### Data collection

Agilent SuperNova Dual diffractometer with Atlas detector
Absorption correction: multi-scan (*CrysAlis PRO*; Agilent, 2010 ▶) *T*_min_ = 0.906, *T*_max_ = 0.967
4743 measured reflections
2258 independent reflections
2134 reflections with *I* > 2σ(*I*)
*R*_int_ = 0.030

#### Refinement

*R*[*F* ^2^ > 2σ(*F* ^2^)] = 0.029
*wR*(*F* ^2^) = 0.074
*S* = 1.08
2258 reflections
194 parameters
2 restraints
H atoms treated by a mixture of independent and constrained refinement
Δρ_max_ = 0.22 e Å^−3^
Δρ_min_ = −0.25 e Å^−3^
Absolute structure: Flack (1983 ▶), 725 Friedel pairs
Flack parameter: −0.05 (6)

### 

Data collection: *CrysAlis PRO* (Agilent, 2010 ▶); cell refinement: *CrysAlis PRO*; data reduction: *CrysAlis PRO*; program(s) used to solve structure: *SHELXS97* (Sheldrick, 2008 ▶); program(s) used to refine structure: *SHELXL97* (Sheldrick, 2008 ▶); molecular graphics: *X-SEED* (Barbour, 2001 ▶) and *DIAMOND* (Brandenburg, 2006 ▶); software used to prepare material for publication: *publCIF* (Westrip, 2010 ▶).

## Supplementary Material

Crystal structure: contains datablock(s) global, I. DOI: 10.1107/S1600536811050446/hg5145sup1.cif

Structure factors: contains datablock(s) I. DOI: 10.1107/S1600536811050446/hg5145Isup2.hkl

Supplementary material file. DOI: 10.1107/S1600536811050446/hg5145Isup3.cml

Additional supplementary materials:  crystallographic information; 3D view; checkCIF report

## Figures and Tables

**Table 1:** Hydrogen-bond geometry (Å, °)

*D*—H⋯*A*	*D*—H	H⋯*A*	*D*⋯*A*	*D*—H⋯*A*
N1—H1⋯O3^i^	0.88 (1)	1.99 (1)	2.833 (3)	163 (3)
C1—H1a⋯O7^ii^	0.98	2.59	3.460 (3)	148
C3—H3a⋯O6^iii^	0.99	2.53	3.502 (3)	169
C3—H3b⋯O2^iv^	0.99	2.42	3.380 (3)	162
C10—H10⋯O5^v^	0.95	2.37	3.223 (3)	149

Symmetry codes: (i) 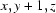; (ii) 
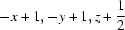
; (iii) 
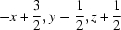
; (iv) 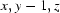; (v) 
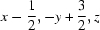
.

**Fig. 1.**
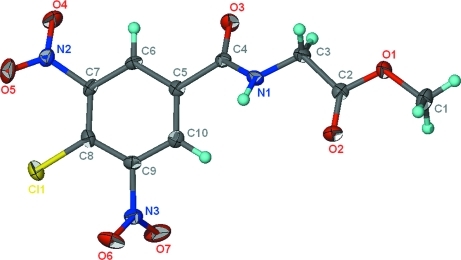
Molecular structure of (I) showing atom-labelling scheme and displacement ellipsoids at the 70% probability level.

**Fig. 2.**
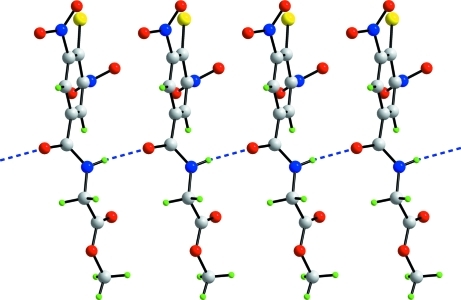
Supramolecular linear chain along the b axis in (I). The N—H···O contacts are shown as blue dashed lines.

**Fig. 3.**
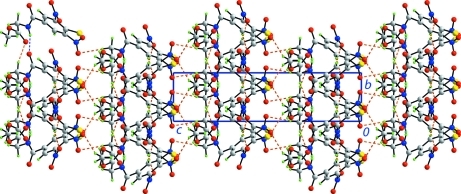
A view of the unit-cell contents of (I) in projection down the a axis. The N—H···O and C—H···O interactions are shown as blue and orange dashed lines, respectively.

## supplementary crystallographic information

### Comment

Molecules related to the title compound, (I), attract interest for their biological properties (Liu *et al.*, 2009) and also in terms of crystal engineering endeavours (Eissmann & Weber, 2011). In (I), Fig. 1, the dihedral angle between the amide (O3,N1,C4) atoms and the benzene ring is 38.75 (11)°. The acetyl group is also twisted out of the plane of the amide group with the C4—N1—C3—C2 torsion angle being -150.1 (2)°. Each nitro group is twisted out of the plane of the benzene ring to which it is connected with the O4—N2—C7—C6 torsion angle = 34.0 (3)° and with O6—N3—C9—C8 = -64.5 (3)°.

The crystal packing is dominated by the formation of linear supramolecular chains along the *b* axis and mediated by N—H···O hydrogen bonds involving the amide group, Fig. 2 and Table 1. Chains are consolidated in the crystal packing by C—H···O interactions, Fig. 3 and Table 1.

### Experimental

To a solution of 4-chloro-3,5-dinitrobenzoic acid (0.48 g, 2 mmol) in dichloromethane (30 ml) was added 1-ethyl-3-(3-dimethylaminopropyl)carbodiimidehydrochloride (0.40 g, 2.1 mmol) and *N*,*N*-dimethylaminopyridine (25 mg, 0.2 mmol). The mixture was stirred at room temperature for an hour. Methyl 2-aminoacetate (178 mg, 2 mmol) in chloroform (20 ml) along with several drops of triethylamine were added. After another six hours, the mixture was subjected to chromatography (petroleum ether/acetone 4:1) to provide the product as a yellow solid (501.5 mg, 80% yield). Crystals were grown from a mixture of dichloromethane and *n*-hexane (1:1 *v*/*v*).

### Refinement

Carbon-bound H-atoms were placed in calculated positions [C—H 0.95 to 0.99 Å, *U*_iso_(H) 1.2 to 1.5*U*_eq_(C)] and were included in the refinement in the riding model approximation. The amino H-atom was located in a difference Fourier map, and was refined with a distance restraint of N—H 0.88±0.01 Å, and with free *U*_iso_.

### Crystal data

**Table:**

C_10_H_8_ClN_3_O_7_	*F*(000) = 648
*M**_r_* = 317.64	*D*_x_ = 1.635 Mg m^−^^3^
Orthorhombic, *P**n**a*2_1_	Mo *K*α radiation, λ = 0.71073 Å
Hall symbol: P 2c -2n	Cell parameters from 2633 reflections
*a* = 14.5219 (5) Å	θ = 2.6–27.5°
*b* = 4.7949 (2) Å	µ = 0.34 mm^−^^1^
*c* = 18.5368 (6) Å	*T* = 100 K
*V* = 1290.74 (8) Å^3^	Prism, yellow
*Z* = 4	0.30 × 0.20 × 0.10 mm

### Data collection

**Table:**

Agilent SuperNova Dual diffractometer with Atlas detector	2258 independent reflections
Radiation source: SuperNova (Mo) X-ray Source	2134 reflections with *I* > 2σ(*I*)
Mirror	*R*_int_ = 0.030
Detector resolution: 10.4041 pixels mm^-1^	θ_max_ = 27.6°, θ_min_ = 2.8°
ω scan	*h* = −13→18
Absorption correction: multi-scan (*CrysAlis PRO*; Agilent, 2010)	*k* = −6→5
*T*_min_ = 0.906, *T*_max_ = 0.967	*l* = −17→24
4743 measured reflections	

### Refinement

**Table:**

Refinement on *F*^2^	Secondary atom site location: difference Fourier map
Least-squares matrix: full	Hydrogen site location: inferred from neighbouring sites
*R*[*F*^2^ > 2σ(*F*^2^)] = 0.029	H atoms treated by a mixture of independent and constrained refinement
*wR*(*F*^2^) = 0.074	*w* = 1/[σ^2^(*F*_o_^2^) + (0.0367*P*)^2^ + 0.1422*P*] where *P* = (*F*_o_^2^ + 2*F*_c_^2^)/3
*S* = 1.08	(Δ/σ)_max_ = 0.001
2258 reflections	Δρ_max_ = 0.22 e Å^−^^3^
194 parameters	Δρ_min_ = −0.25 e Å^−^^3^
2 restraints	Absolute structure: Flack (1983), 725 Friedel pairs
Primary atom site location: structure-invariant direct methods	Flack parameter: −0.05 (6)

### Fractional atomic coordinates and isotropic or equivalent isotropic displacement parameters (Å^2^)

**Table:**

	*x*	*y*	*z*	*U*_iso_*/*U*_eq_	
Cl1	0.97666 (4)	0.68297 (13)	0.49982 (3)	0.02449 (14)	
O1	0.46196 (11)	0.5789 (4)	0.87399 (9)	0.0214 (4)	
O2	0.51448 (12)	0.8605 (3)	0.78629 (9)	0.0232 (4)	
O3	0.73214 (11)	0.1135 (3)	0.76810 (9)	0.0212 (4)	
O4	1.03518 (12)	0.0525 (4)	0.65033 (11)	0.0278 (4)	
O5	1.09547 (11)	0.4311 (4)	0.60728 (11)	0.0282 (4)	
O6	0.81733 (12)	1.0984 (3)	0.50745 (10)	0.0286 (4)	
O7	0.71827 (12)	0.7754 (4)	0.48417 (10)	0.0329 (5)	
N1	0.67321 (13)	0.5506 (4)	0.77382 (11)	0.0154 (4)	
N2	1.03014 (12)	0.2929 (4)	0.62781 (11)	0.0188 (4)	
N3	0.78247 (13)	0.8709 (4)	0.51797 (10)	0.0179 (4)	
C1	0.37848 (17)	0.7447 (6)	0.87927 (14)	0.0260 (5)	
H1A	0.3379	0.6641	0.9160	0.039*	
H1B	0.3469	0.7454	0.8326	0.039*	
H1C	0.3944	0.9363	0.8928	0.039*	
C2	0.52388 (15)	0.6648 (5)	0.82581 (12)	0.0152 (5)	
C3	0.60829 (15)	0.4829 (5)	0.83024 (12)	0.0180 (5)	
H3A	0.6381	0.5085	0.8778	0.022*	
H3B	0.5899	0.2847	0.8259	0.022*	
C4	0.72913 (14)	0.3572 (5)	0.74666 (12)	0.0144 (4)	
C5	0.79056 (16)	0.4510 (5)	0.68634 (11)	0.0141 (5)	
C6	0.87941 (15)	0.3419 (5)	0.68295 (12)	0.0144 (5)	
H6	0.9001	0.2141	0.7186	0.017*	
C7	0.93716 (15)	0.4205 (5)	0.62750 (12)	0.0148 (4)	
C8	0.90958 (15)	0.6012 (5)	0.57315 (12)	0.0154 (5)	
C9	0.81934 (15)	0.6983 (5)	0.57707 (12)	0.0148 (4)	
C10	0.76008 (15)	0.6285 (4)	0.63220 (12)	0.0150 (4)	
H10	0.6992	0.7008	0.6332	0.018*	
H1	0.688 (2)	0.722 (3)	0.7627 (15)	0.034 (8)*	

### Atomic displacement parameters (Å^2^)

**Table:**

	*U*^11^	*U*^22^	*U*^33^	*U*^12^	*U*^13^	*U*^23^
Cl1	0.0208 (2)	0.0329 (3)	0.0197 (3)	−0.0005 (2)	0.0067 (3)	0.0055 (3)
O1	0.0187 (8)	0.0221 (9)	0.0235 (9)	0.0055 (7)	0.0068 (7)	0.0057 (8)
O2	0.0229 (9)	0.0218 (9)	0.0250 (9)	0.0052 (7)	0.0007 (7)	0.0080 (8)
O3	0.0222 (8)	0.0118 (8)	0.0296 (9)	0.0021 (6)	0.0050 (8)	0.0042 (7)
O4	0.0222 (9)	0.0231 (10)	0.0381 (11)	0.0077 (7)	0.0013 (8)	0.0099 (9)
O5	0.0127 (8)	0.0264 (9)	0.0455 (11)	−0.0055 (7)	0.0033 (8)	0.0016 (9)
O6	0.0452 (10)	0.0165 (8)	0.0239 (9)	−0.0042 (8)	−0.0027 (9)	0.0078 (8)
O7	0.0313 (10)	0.0340 (11)	0.0334 (11)	−0.0022 (8)	−0.0180 (9)	0.0075 (9)
N1	0.0197 (9)	0.0095 (9)	0.0171 (9)	0.0005 (7)	0.0019 (8)	0.0020 (8)
N2	0.0140 (10)	0.0219 (11)	0.0206 (10)	−0.0005 (8)	−0.0006 (8)	0.0001 (9)
N3	0.0220 (9)	0.0187 (10)	0.0129 (9)	0.0047 (8)	0.0004 (8)	−0.0007 (8)
C1	0.0173 (11)	0.0303 (13)	0.0305 (13)	0.0060 (11)	0.0032 (11)	−0.0036 (13)
C2	0.0167 (10)	0.0155 (11)	0.0133 (11)	0.0003 (9)	−0.0011 (9)	−0.0038 (9)
C3	0.0195 (11)	0.0176 (12)	0.0171 (11)	0.0029 (9)	0.0023 (9)	0.0044 (9)
C4	0.0133 (9)	0.0149 (12)	0.0150 (10)	−0.0016 (8)	−0.0039 (9)	0.0012 (9)
C5	0.0152 (10)	0.0128 (11)	0.0142 (10)	−0.0023 (9)	−0.0013 (8)	−0.0019 (9)
C6	0.0158 (11)	0.0112 (11)	0.0161 (10)	0.0023 (9)	−0.0031 (9)	0.0005 (9)
C7	0.0111 (10)	0.0131 (10)	0.0201 (11)	0.0018 (9)	−0.0011 (9)	−0.0035 (9)
C8	0.0148 (10)	0.0163 (12)	0.0149 (10)	−0.0029 (9)	0.0026 (9)	−0.0019 (9)
C9	0.0186 (11)	0.0106 (11)	0.0151 (10)	0.0000 (9)	−0.0016 (9)	0.0011 (9)
C10	0.0154 (10)	0.0113 (10)	0.0185 (11)	0.0002 (9)	0.0001 (9)	−0.0029 (9)

### Geometric parameters (Å, °)

**Table:**

Cl1—C8	1.718 (2)	C1—H1B	0.9800
O1—C2	1.333 (3)	C1—H1C	0.9800
O1—C1	1.453 (3)	C2—C3	1.507 (3)
O2—C2	1.198 (3)	C3—H3A	0.9900
O3—C4	1.235 (3)	C3—H3B	0.9900
O4—N2	1.228 (3)	C4—C5	1.499 (3)
O5—N2	1.218 (2)	C5—C10	1.388 (3)
O6—N3	1.218 (2)	C5—C6	1.394 (3)
O7—N3	1.213 (2)	C6—C7	1.379 (3)
N1—C4	1.331 (3)	C6—H6	0.9500
N1—C3	1.445 (3)	C7—C8	1.388 (3)
N1—H1	0.875 (10)	C8—C9	1.393 (3)
N2—C7	1.482 (3)	C9—C10	1.377 (3)
N3—C9	1.474 (3)	C10—H10	0.9500
C1—H1A	0.9800		
			
C2—O1—C1	116.05 (18)	C2—C3—H3B	109.4
C4—N1—C3	121.01 (19)	H3A—C3—H3B	108.0
C4—N1—H1	115 (2)	O3—C4—N1	124.0 (2)
C3—N1—H1	123 (2)	O3—C4—C5	120.2 (2)
O5—N2—O4	124.78 (19)	N1—C4—C5	115.9 (2)
O5—N2—C7	118.92 (19)	C10—C5—C6	119.5 (2)
O4—N2—C7	116.30 (18)	C10—C5—C4	122.2 (2)
O7—N3—O6	125.1 (2)	C6—C5—C4	118.15 (19)
O7—N3—C9	116.80 (19)	C7—C6—C5	119.6 (2)
O6—N3—C9	118.10 (19)	C7—C6—H6	120.2
O1—C1—H1A	109.5	C5—C6—H6	120.2
O1—C1—H1B	109.5	C6—C7—C8	122.43 (19)
H1A—C1—H1B	109.5	C6—C7—N2	115.99 (19)
O1—C1—H1C	109.5	C8—C7—N2	121.56 (19)
H1A—C1—H1C	109.5	C7—C8—C9	116.3 (2)
H1B—C1—H1C	109.5	C7—C8—Cl1	123.59 (17)
O2—C2—O1	125.1 (2)	C9—C8—Cl1	119.92 (18)
O2—C2—C3	125.4 (2)	C10—C9—C8	123.1 (2)
O1—C2—C3	109.49 (19)	C10—C9—N3	117.46 (19)
N1—C3—C2	111.18 (18)	C8—C9—N3	119.4 (2)
N1—C3—H3A	109.4	C9—C10—C5	119.1 (2)
C2—C3—H3A	109.4	C9—C10—H10	120.5
N1—C3—H3B	109.4	C5—C10—H10	120.5
			
C1—O1—C2—O2	−1.9 (3)	O4—N2—C7—C8	−144.0 (2)
C1—O1—C2—C3	176.48 (19)	C6—C7—C8—C9	−0.6 (3)
C4—N1—C3—C2	−150.1 (2)	N2—C7—C8—C9	177.3 (2)
O2—C2—C3—N1	−7.6 (3)	C6—C7—C8—Cl1	−174.96 (18)
O1—C2—C3—N1	174.00 (19)	N2—C7—C8—Cl1	3.0 (3)
C3—N1—C4—O3	−2.2 (3)	C7—C8—C9—C10	1.7 (3)
C3—N1—C4—C5	177.61 (19)	Cl1—C8—C9—C10	176.32 (18)
O3—C4—C5—C10	139.5 (2)	C7—C8—C9—N3	−174.6 (2)
N1—C4—C5—C10	−40.3 (3)	Cl1—C8—C9—N3	−0.1 (3)
O3—C4—C5—C6	−36.8 (3)	O7—N3—C9—C10	−59.9 (3)
N1—C4—C5—C6	143.4 (2)	O6—N3—C9—C10	118.9 (2)
C10—C5—C6—C7	2.6 (3)	O7—N3—C9—C8	116.6 (2)
C4—C5—C6—C7	179.0 (2)	O6—N3—C9—C8	−64.5 (3)
C5—C6—C7—C8	−1.6 (3)	C8—C9—C10—C5	−0.7 (3)
C5—C6—C7—N2	−179.6 (2)	N3—C9—C10—C5	175.76 (19)
O5—N2—C7—C6	−144.8 (2)	C6—C5—C10—C9	−1.5 (3)
O4—N2—C7—C6	34.0 (3)	C4—C5—C10—C9	−177.75 (19)
O5—N2—C7—C8	37.1 (3)		

### Hydrogen-bond geometry (Å, °)

**Table:**

*D*—H···*A*	*D*—H	H···*A*	*D*···*A*	*D*—H···*A*
N1—H1···O3^i^	0.88 (1)	1.99 (1)	2.833 (3)	163 (3)
C1—H1a···O7^ii^	0.98	2.59	3.460 (3)	148
C3—H3a···O6^iii^	0.99	2.53	3.502 (3)	169
C3—H3b···O2^iv^	0.99	2.42	3.380 (3)	162
C10—H10···O5^v^	0.95	2.37	3.223 (3)	149

Symmetry codes: (i) *x*, *y*+1, *z*; (ii) −*x*+1, −*y*+1, *z*+1/2; (iii) −*x*+3/2, *y*−1/2, *z*+1/2; (iv) *x*, *y*−1, *z*; (v) *x*−1/2, −*y*+3/2, *z*.
